# The application of new breeding technology based on gene editing in pig industry — A review

**DOI:** 10.5713/ab.21.0390

**Published:** 2022-01-05

**Authors:** Ching-Fu Tu, Chin-kai Chuang, Tien-Shuh Yang

**Affiliations:** 1 Division of Animal Technology, Animal Technology Research Center, Agricultural Technology Research Institute, Hsinchu City 30093, Taiwan; 2 Department of Biotechnology and Animal Science, National Ilan University, Yilan City, Yilan County 26047, Taiwan

**Keywords:** CRISPR/Cas9, Genome/Gene-editing (GE), New Breeding Technique (NBT), Pig, Regulation

## Abstract

Genome/gene-editing (GE) techniques, characterized by a low technological barrier, high efficiency, and broad application among organisms, are now being employed not only in medical science but also in agriculture/veterinary science. Different engineered CRISPR/Cas9s have been identified to expand the application of this technology. In pig production, GE is a precise new breeding technology (NBT), and promising outcomes in improving economic traits, such as growth, lean or healthy meat production, animal welfare, and disease resistance, have already been documented and reviewed. These promising achievements in porcine gene editing, including the Myostatin gene knockout (KO) in indigenous breeds to improve lean meat production, the uncoupling protein 1 (*UCP1*) gene knock-in to enhance piglet thermogenesis and survival under cold stress, the generation of GGTA1 and CMP-N-glycolylneuraminic acid hydroxylase (*CMAH*) gene double KO (dKO) pigs to produce healthy red meat, and the KO or deletion of exon 7 of the *CD163* gene to confer resistance to porcine reproductive and respiratory syndrome virus infection, are described in the present article. Other related approaches for such purposes are also discussed. The current trend of global regulations or legislation for GE organisms is that they are exempted from classification as genetically modified organisms (GMOs) if no exogenes are integrated into the genome, according to product-based and not process-based methods. Moreover, an updated case study in the EU showed that current GMO legislation is not fit for purpose in term of NBTs, which contribute to the objectives of the EU’s Green Deal and biodiversity strategies and even meet the United Nations’ sustainable development goals for a more resilient and sustainable agri-food system. The GE pigs generated via NBT will be exempted from classification as GMOs, and their global valorization and commercialization can be foreseen.

## INTRODUCTION

Current intensive systems of livestock production are major contributors to environmental degradation leading to climate change, biodiversity losses, and animal welfare and even human health concerns. Therefore, so-called sustainable animal husbandry must be managed in a smart way to achieve not only production value but also of planet and people benefits. From the perspective of pig rearing, systemic selection for body length, backfat thickness, and growth has been applied since the 1920s [1]; however, some undesirable side effects, such as leg disorders, physiological unfitness and susceptibility to stress or disease, have concomitantly resulted from selection for high production efficiency [2,3]. New phenotypes have been proposed to meet sustainable breeding goals in pigs [4], and selection for productivity and robustness traits in pigs has become an issue [5].

The advance of molecular biology technology has enabled breeding for a specific trait  possible through genomic selection, and the use of gene-editing technology can even introduce targeted traits in pigs [6]. New breeding technology (NBT) presents promise not only in agriculture but also in biomedicine [7]. The implementation of NBT with the goal of robustness may be learnt by naturally selected fitness since random changes in genetic information that are most likely to benefit survival, reproduction, and adaptability to the environment are the mechanisms driving this revolution. In contrast to natural selection, artificial selection is a practice used by humans to develop new organisms with desirable traits; in agriculture, it has yielded different outcomes, including both gains and losses of diversity. Genetic variation among crop breeds has been increasing due to the *in vitro* mutagenesis approach since 1930, but this is less the case in farm animals because the proliferation of stocks with phenotypic traits of commercial value has gradually homogenized their genetic background. Once wild-type alleles are eliminated from the population, livestock become dependent on farm feeding for their survival. In the case of pigs, many indigenous breeds are being lost, as native breeds are survival driven, and their environmental fitness shows little association with phenotypes related to economic viability, which has been the chief goal of commercial pig breeding [8]. The application of NBT means that pigs can be selectively bred for utility in both natural and artificial environments (i.e., sustainable production value) based on human design as the dominant factor [8].

The conservation of biodiversity is not only ethical but also practical, especially in crucial environmental gradients. For example, indigenous breeds may be adapted to a special environment, whether biological or physical (e.g., tolerance to African swine fever (ASF) infection in warthog [9] and heat tolerance in ethnic cattle [10]). The power of whole-genome sequencing may reveal the genetic source of these valuable features and offer opportunities to search for them in wild-type species, and the results can be referenced and used for improving the performance of commercial breeds.

Genome/gene-editing (GE) techniques include zinc finger nuclease (ZFN)-, transcription activator-like effector nuclease (TALEN)-, and clustered regularly interspaced short palindromic repeat (CRISPR)/CRISPR-associated (Cas) endoribonuclease 9 (Cas9)-based methods. The CRISPR/Cas9 method has become the dominant approach since it is characterized by a low technological barrier and high efficiency; its application in pigs has been recently reviewed [11,12], and its use to improve resilience/disease resistance with the goal of epidemic elimination is of particular interest [13]. In pig production, GE has been recognized as a precise NBT with promising outcomes in improving sustainable traits; although this has already been documented, the predicted valorization and commercialization of NBT for public use remain uncertain considering related legislation. The present review will briefly introduce the current progress in NBT, followed by the application of NBT to pig production to obtain healthy pork and achieve disease resilience. Additionally, regulatory concerns will be briefly discussed to understand the controversy regarding NBT-derived products.

## GENOME/GENE-EDITING TECHNOLOGY: AN OVERVIEW

To date, three GE technologies, based on ZFN [14], TALEN [15], and CRISPR/Cas9 [16], have been developed. Currently, CRISPR/Cas9 is regarded as a canonical methodology and a precise NBT with high efficiency and rapid realization, a low technological barrier and low cost that can be broadly applied in many organisms, including the livestock.

**Figure 1:**
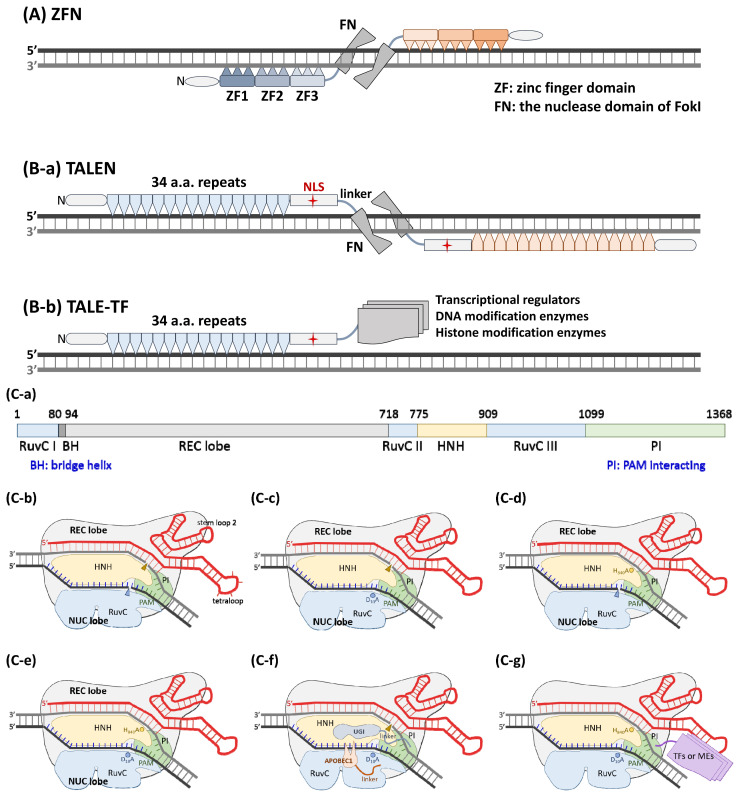
Genome/gene-editing nucleases. (A) Illustration of a pair of functional ZFNs bound to DNA. An N-terminal domain is shown to aid in the folding of zinc finger domains. Each tri-ZF is fused with the nuclease domain of FokI (FN) via a peptide linker. The recognition sites of each pair of ZFNs are organized in a tail-to-tail orientation to perform effective double-strand cutting activity. (B-1) A model consisting of a pair of TALENs in a head-to head orientation is shown. An N-terminal domain is also needed to facilitate the folding of the 34 AA repeat domains. A C-terminal domain containing an NLS is essential for enzyme activity. (B-2) The engineered TALE can be used as a sequence-specific DNA binding domain to carry a transcriptional regulator, DNA-modifying enzyme, or histone modification enzyme to the DNA region of interest. The domain organization of SpCas9 (C-a) and a schematic diagram of wild-type SpCas9 associated with a sg-RNA (C-b) are shown. (C-c) The noncomplementary strand is cut by the RuvC nuclease domain, and this nuclease activity is blocked in the D10A mutant. (C-e) The complementary strand is digested by the HNH nuclease domain, and this nuclease activity is blocked in the H840A mutant. (C-f) Both nuclease activities of SpCas9 are lost in the D10A/ H840A double mutant, which is referred to as dead Cas9 (dCas9). (C-d) The D10A mutant, also known as Cas9 nickase (nCas9), is engineered as a C to T nucleotide editor by linking a cytidine deaminase, APOBEC1, to its N-terminus, and the switching probability can be increased by the fusion of a uracil glycosylase inhibitor (UGI) to the C-terminus of nCas9. (C-g) Similar to TALE, dCas9 can be guided by a sgRNA as a sequence-specific DNA-binding roboprotein. Transcriptional regulators, DNA-modifying enzymes, or histone-modifying enzymes can be fused to either or both of the N- and C-termini.

Considering that the genome sizes of mammalian and bird cells per ploidy are approximately one to three billion base pairs (bp), the length of a DNA sequence that theoretically appears only once in the genome should be at least 16 bp (4^n^≥3E9, n≥16). Initially, natural DNA-binding proteins were screened to identify those that can recognize a stretch of DNA longer than 16 bp, and homing endonucleases (HEs), which display an economy of size and yet recognize long DNA sequences (typically 20 to 30 base pairs) [17], were employed for this purpose. The number of HE members in a collection should be billions to cover the whole mammalian genome, which is an unreachable goal. A modular C2H2 zinc finger (ZF) recognizes a sequence of 3 bp, therefore, a collection of 64 ZFs can represent all 3 bp combinations. Usually, a pair of triZFs are engineered and recruited to bind two adjacent 9-bp DNA stretches. Each triZF is fused with the nuclease domain of type IIS FokI (FN) to serve as the ZFN. The nuclease activity of FN exclusively appears in its dimeric form Smith et al [18]. A pair of ZFNs with recognition sites in a tail-to-tail orientation was demonstrated to be necessary for effective double-strand cutting activity [19] (Figure 1A).

The bacterial transcription activator-like effector (TALE) protein contains an array of 34 amino acid (AA) repeats, each of which recognizes a bp sequence via the 12th and 13th residues, known as the repeat variable di-residue (RVD), together. More simply, only 4 kinds of repeats, each carrying an RVD for distinguishing G, A, T and C, are sufficient building blocks for any DNA sequence [20,21]. It is feasible to assemble more than 20 repeats into an engineered TALE corresponding to the target DNA sequence of interest via the Golden Gate cloning method in a few days [22–25]. Similar to the ZFN-based method, a pair of TALENs (TALE fused with FN at the C-terminus) specifically recognize sites in a head-to-head orientation and cause a double-strand break (Figure 1B-a). In addition to the FN nuclease domain, the transcription regulatory domain and DNA-modifying enzymes can be engineered at the C-terminus of the sequence-specific TALE core structure to create artificial gene-editing factors [26,27] (Figure 1B-b).

Both ZF and TALE use AA residues to recognize nucleotides, and intrinsic limitations regarding specificity and off-targeting are unavoidable. The breakthrough CRISPR/Cas9 technology, in which a single-guide RNA (sgRNA), an artificial fusion of crRNA and tracrRNA, is used to distinguish target DNA sequences via a Watson-Crick base-pairing mechanism with precise specificity to prevent off-targeting problem, was first reported in 2012 [28]. The target site, a protospacer matched to the spacer portion of the guide RNA and a protospacer adjacent motif (PAM), and its counterpart Cas9/sgRNA complex first interact between the PAM and PI (PAM interacting) domains. This event causes the target DNA double helix to bend to allow a melted region, where the spacer RNA/target strand DNA heteroduplex begins and extends, to form an R-loop, which can induce the conformation of the Cas9 protein to shift to a nuclease-activated state [29,30]. The target-strand DNA and non-target-strand DNA sequences are independently cleaved by the His-Asn-His motif containing endonuclease (HNH) and resistance to UV light-C (RuvC) nuclease domains of Cas9, respectively (Figure 1C-1a,b). Two types of DNA modification processes, nonhomologous end joining and homology-directed recombination, have been widely used for genome/gene editing [31]. The D_10_A [28] (Figure 1C-c) and H_840_A [28] mutations (Figure 1C-e) and the N_863_A [32] mutation independently destroy the nuclease activity of the RuvC and HNH domains, respectively; however, they do not influence target site binding affinity. Cas9 carrying either one or two of these mutations is referred to as Cas9 nickase (nCas9) or the dead Cas9 enzyme (dCas9) (Figure 1C-f), respectively. To prevent unwanted indel mutations or usage of donor template DNA, nCas9 and dCas9 are utilized as a DNA-targeting core loaded with cytosine deaminase [33], adenine deaminase [34,35], an uracil glycosylase inhibitor [36] (Figure 1C-d), or reverse transcriptase [37,38] (Figure 1C-g) to perform more precise and versatile genome/gene editing.

## APPLICATION OF NEW BREEDING TECHNOLOGY IN PIG PRODUCTION

### Lean growth promotion

A natural mutation of the myostatin (*MSTN*) gene in some cattle breeds results in double muscling [39,40]. Czaja et al [41] recently revealed that MSTN may regulate pituitary development and function and that its inhibitory actions in muscle may be partly mediated by attenuating growth hormone action in the liver, leading to the expression of insulin-like growth factor 1 (IGF 1). In pigs, MSTN, IGF2 repressor Zinc finger BED-type containing 6 (ZBED6) and *Fbox* protein 40 (*Fbox* 40) gene knockout (KO) can improve growth or muscle mass production, but Iroquois homeobox 3 (IRX3) KO does not have this effect, as summarized in Table 1.

The first successful case of pig *MSTN* KO was achieved by using ZFN in the Meishan breed, and the resultant homozygotes reached adulthood normally but showed a higher percentage of lean body mass growth, exhibiting wider dorsal musculature and double muscling of the hip in particular [42]. However, the same attempt to achieve *MSTN* gene KO by using CRISPR/Cas9 failed to produce healthy KO piglets in the Landrace and Large White breeds [43,44]; but they later successfully generated 23 Erhualian ethnic breed pigs showing obvious muscular protrusion, a wider back and fuller hips relative to non-KO pigs [46]. Since Wang et al [45] suggested that commercial breeds would be more sensitive to the KO of endogenous genes, Zou et al [46] generated two healthy Duroc pigs with the Belgian Blue mutation only in heterozygosity, but they did not show double muscling at the neonatal stage. It could be explained by a recent finding that showed in commercial *MSTN* KO pigs, the decreased expression level of type I collagen and Scleraxis, could result in umbilical hernia and tippy-toe standing problems typified by the tendon and linea alba dysfunction [47].

Similarly, using CRISPR/Cas9, Xiang et al [48] mutated the *IGF2* intron 3–3072 site of the *IGF2* gene, which abolished *ZBED6* repressor binding, causing the loss of its regulatory function, in indigenous Bama minipigs and obtained healthy animals with an improved growth rate, higher lean content, and little change in meat quality. This was the first report that editing noncoding regions could improve economic traits in pigs of indigenous breeds. Furthermore, Li et al [49] used CRISPR/Cas9 editing and introduced two mutations (PVD20H and GP19del) in the MSTN signal peptide region in Liang Guang Small Spotted pigs, an indigenous Chinese breed, without inhibiting mature MSTN production. This approach downregulated *MSTN*^+/PVD20H^ and *MSTN*^KO/PVD20H^ and upregulated myogenic regulatory factors, including MyoD, Myo-genin, and Myf-5. The precise editing of the MSTN signal peptide enhanced porcine muscle development without markedly affecting the expression of the mature MSTN peptide. This might be a better KO approach applied to commercial breeds for the further improvement of lean body mass without disturbing their normal physiology, such as causing reproductive disorders or increasing stress susceptibility, as observed in the highly muscled pig breed Pietrain.

*Fbox* 40 in mice targets insulin receptor substrate 1 (IRS1) for ubiquitylation and degradation, and the abnormal expression of *FBOX* 40 in humans is associated with muscle pathology, causing limb-girdle muscle dystrophy [50]. Zou et al [51] performed Neo resistance gene knock-in (KI) into exon 4 of *FBOX* 40 to abolish its gene function and identified increased IRS1 expression and a 4% increase in muscle mass growth. The authors recapitulated human muscular disease and suggested the application of this strategy in pig production. Similarly, the *IRX3* gene is implicated in human obesity and controls body mass and body composition in mice, but Zhu et al [52] found that clones of *IRX3*^−/−^ Bama minipigs showed a significantly decreased birth weight, poor viability, and short survival after farrowing. The disparity in these results might originate from errors in somatic cell nuclear transfer (SCNT), although the authors hypothesized that *IRX3* may be responsible for some important physiological functions in pigs and should not be targeted as a gene-editing candidate for body fat reduction [52].

### Improvement of thermogenesis in piglets cold stressed

Domestic pigs have no brown adipose tissue (BAT) and show no uncoupling protein 1 (UCP1) expression in their mitochondria [53,54], and exons 3 to 5 of the *UCP1* gene were deleted during evolution 20 million years ago [55]. The function of UCP1 is to disengage oxidative phosphorylation from ATP synthesis in mitochondria and dissipate energy as heat in BAT for survival in cold environments or under cold stress. Although Lin et al [56] proved that cold adaption in pigs depends on UCP3 in beige adipocytes, pigs show poor thermoregulation [55] due to the absence of nonshivering thermogenesis; thus, high thermoneutrality is needed. The provision of additional warmth by various means to keep piglets warm is a basic practice of producers, especially in temperate regions. Zhang et al [57] constructed a porcine adiponectin promotor with mouse *UCP1* cDNA and performed KI of the exogene at the porcine *UCP1* exon 2 site through GE by using CRISPR/Cas9. The *UCP1* KI piglets could maintain a normal rectal temperature of 38°C during 4 h of 4°C cold exposure, whereas the control group showed hypothermia (2°C lower). Since the adiponectin promoter drove *UCP1* expression in adipocytes, the KI pigs grew normally relative to the control pigs, without noticeable harm to their well-being during the 6-month study period. Carcass evaluation further showed that backfat thickness, adipocyte size, and body fat accretion were all significantly decreased, resulting in an improvement in the carcass lean percentage. The authors also claimed that KI pigs showed a loss of body fat upon UCP1 activation in white adipocyte tissues, which would further improve pig welfare and reduce economic losses due to external energy expenditure to achieve thermoneutrality, especially in post farrowing piglets.

### Healthy pork production

Red meat (pork, beef, and mutton) consumption may induce allergies due to the presence of alpha-Gal on the muscle cell surface [58]. This uncommon alpha-gal syndrome [59] can be avoided by choosing a product from which the antigen has been removed in KO livestock. Recently, the FDA approved the commercialization of *GGTA1* gene-edited or KO pigs, also known as GalSafe pigs, for pork consumption [60]. This is the first case of a livestock biotechnology product launched for both food and biomedical uses.

The red meat cell surface expresses tremendous amounts of N-glycolylneuraminic acid (Neu5Gc; nonhuman glycan) [61], which, once absorbed, is incorporated on the surface of human cells [62] and elicits chronic inflammation, causing a major risk of colorectal cancer and atherosclerosis development [63]. Transforming Neu5Gc to Neu5Ac (the human form) in pigs through the KO of the responsible CMP-N-glycolylneuraminic acid hydroxylase (*CMAH*) gene has been a goal of xenotransplantation, and in animal production, this would transform red meat into white since poultry and fish also show the Neu5Ac form. Our research group has generated alpha-gal [64] and Neu5Gc [65] KO pigs by CRISPR/Cas9 GE. Through crossbreeding, dKO offspring without any major pathophysiological indications were obtained. After comparing the intestinal decellular scaffold (extracellular matrix, ECM) from dKO and wild-type (WT) pigs, we implanted dKO or WT ECM into dKO recipient longissimus and found that the dKO ECM evoked less inflammation than the WT ECM [66]. It is suggested that the dKO pigs will provide better medical grafts than WT pigs and that the dKO pigs can be considered an animal model for studies of alpha-Gal allergy and may also recapitulate human sialic biology to a greater extent in both healthy and diseased conditions [67]. Additionally, these dKO pigs could be served as a heathy red meat source.

Recent efforts of exogenes KI at *Rosa26* locus by CRISPR/Cas9, healthy and tasty pork could also be resulted [68,69]. You et al [68] generated dual, *Fat1* and *IGF-1*, transgenic (TG) pigs that could provide pork with a significantly higher ω-3 polyunsaturated fatty acid (PUFA) level and a significantly lower ratio of ω-6 PUFA/ω-3 PUFA. Gu et al [69] generated TG pigs carrying muscle-specific overexpression of peroxisome proliferator-activated receptor gamma2 (PPARγ2), which significantly increased intramuscular fat content while maintaining carcass lean ratio. Although, KI the *PPARγ2* gene is considered TG in pigs, yet, both sequences of promoter and *PPARγ2* gene are based on pig genome [68] and thus the transgenes are “safe-harbored” in *Rosa26* locus, making the pork safe as the ordinary.

## APPLICATION OF NEW BREEDING TECHNOLOGY FOR PIG DISEASE TOLERANCE OR RESISTANCE

Genetic editing is vital for a proper understanding of disease mechanisms. Basically, virions infect pigs by contacting receptors on the surface of target cells and then enter the infected cells (e.g., macrophages) via pinocytosis. In theory, deleting the binding domain would disable the virion receptor, an approach that can only be effectively achieved by GE, thereby infection could be avoided. To date, gene editing has been applied in pig breeding to achieve resistance against diseases including porcine reproductive and respiratory syndrome virus (PRRSV) [70–76], African swine fever virus (ASFV) [77,78], porcine epidemic diarrhea virus (PEDV) [65,79], transmissible gastroenteritis virus (TGEV) [75,79] and classical swine fever virus (CSFV) [80], with encouraging findings (Table 2).

### Porcine reproductive and respiratory syndrome virus resistance

PRRSV emerged in the late 1980s and rapidly became an epidemic devastating the pig industry globally. *In vivo*, the virus shows very narrow cell tropism, targeting specific subsets of porcine monocytes/macrophages, and it infects the cells via the heparan sulfate, sialoadhesin (CD169) and CD163 receptors [81]. The CD169 KO pigs generated via traditional homologous recombination and SCNT were not resistant to PRRSV infection, suggesting that CD169 is not necessary for PRRSV infection [82]. To date, CD163 on porcine macrophages has been the best-studied receptor involved in PRRSV infection [83]. Efforts including the KO of CD163 [70,71,75,76], deletion of exon 7 (scavenger receptor cysteine-rich domain 5 region of the CD163 protein) of the CD163 gene [71,74], and the deletion of a portion of exon 7 in the infective pocket of virons [73] have achieved full resistance to PRRSV infection without disturbing the well-being of GE pigs [72–74,84–86]. Furthermore, Xu et al [75] generated dKOs of CD163 and *pAPN* (porcine aminopeptidase N, a factor responsible for TGEV infection) and proved that dKOs pigs could be resistant to type II PRRSV and TGEV infection.

### Challenges of African swine fever virus

When infected by ASFV, domestic pigs and Eurasian wild boars (*Sus scrofa*) develop a lethal hemorrhagic fever, whereas this is not observed in warthogs (*Phacochoerus africanus*) or bush pigs (*Potamochoerus larvatus*), which do not develop marked clinical signs; the last two species evolved in southeastern Africa in a sylvatic cycle with a vector of ASFV the common and argasid ticks of the *Ornithodoros moubata* species complex that live in their burrows and in the pens of domesticated pigs. ASFV has gradually became widespread in eastern and central Europe while showing much faster outbreaks in China and other countries in Asia [8]. Macrophages have been identified as the target cells of ASFV [87], and antibodies against CD163 are able to inhibit both ASFV infection and viral particle binding to alveolar macrophages, highlighting the role of this molecule as a putative receptor for the virus [88]. However, Popescu et al [78] challenged CD163 KO pigs with the Georgia 2007/1 ASFV isolate and failed to reveal any resistance to viral infection, and the observed clinical signs, mortality, pathology, and viremia differed little between KO and WT pigs. A group at the University of Edinburgh has identified polymorphic variation in *RELA* (p65; v-rel reticuloendotheliosis viral oncogene homolog A), the major component of the NF-κB transcription factor and revealed that three AAs of RELA differ between warthogs and domestic pigs [89]. Subsequently, they generated live *RELA* KO pigs by TALEN and ZFN editing [90] and substituted three AAs of domestic pig RELA with warthog AAs by using CRISPR/Cas9 [77]. However, the same research team recently proved that the 3 AAs substitution was not sufficient to confer resilience to ASFV; it only delayed the onset of clinical symptoms and resulted in less virus in nasal secretions and blood in some animals [91]. On the other hand, a German group transfected wild boar lung cell lines with a plasmid encoding Cas9 and a guide RNA targeting codons 71 to 78 of the phosphoprotein *p30* gene (*CP204L*) of ASFV and demonstrated that ASFV plaque formation was completely abrogated, and virus yields were reduced by four orders of magnitude due to targeted Cas9 cleavage of the virus genome [92]. In such an approach, Cas9 and the guide RNA plasmids need to be integrated into the cell genome to maintain long-term resistance to ASFV infection. Thus, once the cells are cloned into animals, they might be argued to be GMOs with a high probability of off-target effects due to the integration and expression of the *Cas9* gene. Recently, Xie et al [80] used a CRISPR/Cas9-mediated KI strategy to generate TG pigs carrying antiviral small hairpin RNAs (shRNAs), safely integrated at the porcine *Rosa26* (*pRosa26*) locus, to test the resistance to anti-CSFV. They found that in the TG pigs subjected to *in vitro* or *in vivo* CSFV challenge, the replication of CSFV was effectively limited, as demonstrated by reduced CSFV-associated clinical symptoms and mortality. Furthermore, this disease resistance could be stably transmitted to the F1 generation. A similar approach should effectively generate ASFV-resistant pigs, but the outcome remains to be seen.

### Coronavirus tolerance in pigs

Four genera of coronaviruses (CoVs), including α-, β-, γ-, and δ-coronaviruses, have been identified. Currently, six CoVs are known to infect pigs, including four α-coronaviruses (TGEV, porcine respiratory coronavirus [PRCV], PEDV, and swine acute diarrhea syndrome-coronavirus [SADS-CoV]), one β-coronavirus (porcine hemagglutinating encephalomyelitis virus [PHEV]), and one porcine δ-coronavirus (PDCoV), which cause different types of infections of great commercial concern in pigs [93]. Among these viruses, TGEV, PRCV, and PHEV have globally circulated in pig populations for decades with few clinical effects, whereas PEDV, PDCoV, and SADS-CoV are considered emerging CoVs and cause severe acute diarrhea in piglets with high mortality. These viruses infect host target cells via S-protein binding to the proposed receptors of aminopeptidase N (APN) and Neu5Gc; however, porcine GE by CRISPR/Cas9 for *APN* [76,79] or *CMAH* [65] KO did not achieve PEDV infection resistance, indicating that these receptors might not be sufficient for PEDV infection. However, *APN* KO pigs could resist TGEV infection [75,79] and showed decreased susceptibility to PDCoV infection with normal growth performance [75]. CoVs are RNA^+^ viruses, and their S-protein is a glycoprotein that undergoes complicated posttranslational modifications to achieve diverse antigenicity [94]. These highly variable sequences [95] hinder effective vaccine development.

### Other viral disease

The challenges of precisely editing the genome, developing effective vaccines, and designing a strategy ensuring biosecurity in the face of ASFV and CoVs threats remain in pig production. As mentioned above, Xie et al [80] performed the CRISPR/Cas9-mediated knock-in of anti-CSFV antiviral shRNAs and achieved effective CSFV infection resistance. Using this TG strategy, Hu et al [96] generated TG pigs constitutively expressing FMDV-specific shRNAs and found that these animals showed higher resistance to FMDV. It appears that combining shRNA and GE to target and degenerate the critical region of the virus genome, without the integration of the *Cas9* gene, could be a viable strategy for achieving resistance in animals to lessen or even prevent infection.

## REGULATORY ISSUES

Although GE creates variants with indel mosaicism and may generate off-target effects, the CRISPR/Cas9 system is a canonical technology with high efficiency, fast performance, a low technological barrier, and low cost and can be broadly applied to many organisms. However, there is still debate concerning the organisms or products generated via GE, as genetically modified organisms (GMOs) are subject to diverse regulations globally. For example, based on the definition of not carrying exo-nucleic acids, Argentina, Austria, Brazil, Canada, Chile, and Japan exempt these organisms from classification as GMOs [97,98]. In the USA, although the title of Guidance for Industry 187 has been changed from “Guidance for Industry on Regulation of Genetically Engineered Animals Containing Heritable Recombinant DNA Constructs” (2015 revision) to “Regulation of Intentionally Altered Genomic DNA in Animals” (2017 draft), but the marketing of GE animals, their offspring, and their food products (milk, meat, and eggs) is not allowed before obtaining the approval of a New Animal Drug Application (NADA) granted by the FDA. Furthermore, on July 28th, 2018, the European Court of Justice (ECJ) issued a directive stating that organisms obtained via directed mutagenesis techniques (genome editing) are regarded as GMOs because their genome had been altered (according to a process-based principle). The potential benefits of gene editing for the future of agriculture are well covered, and the regulatory constraints that limit the ability to maximize their objective function can be considered in relation to financial returns. For instance, a 15-year delay in the introduction of PRRSV-resistant pigs to the USA and EU would cause the loss of $28.3 billion USD [99]. Furthermore, legislative restrictions discourage the valorization and commercialization of NBT-based innovations and overlook the new opportunities they provide regarding not only food security but also biosafety in less-developed regions or underprivileged communities.

Since 1930, many agricultural varieties have been generated via *in vitro* mutagenesis together with phenotypic selection and broadly used in EU agricultural production; however, the EU considers these organisms to be non-GMOs based on their long history of safe use in the food chain. Because the genomes all varieties generated by *in vitro* mutagenesis have been altered, they should be classified as GMOs according to process-based principles, and the situation for GE organisms is similar. Recently, a case study from the EU showed that current GMO legislation is not fit for purpose in term of new genomic techniques (NGT), which alters the genome of an organism [100]. Furthermore, they claim that several plant products obtained via NGT contribute to the objectives of the EU’s Green Deal and biodiversity strategies and even meet the United Nations’ sustainable development goals for a more resilient and sustainable agri-food system. However, the case study still is being discussed by EU ministers in the Agriculture and Fisheries Council as of 2021 and remains to be addressed by the ECJ. Some new guidelines may be added to direct the legal system and allow NGT (or NBT) to contribute to a new era of EU farming.

All GE organisms should be evaluated based on their phenotype and derived products and through scientific comparison to products currently available on the market. GE animals and products should be exempted from classification as GMOs or GM products and allowed to be used in the food production system.

## PERSPECTIVES

The GE animals may show new genotype – phenotype associations; after all an optimal genome expression may not necessarily result in optimal phenotypes. A quantitative genetic analysis is essentially needed to obtain information on the interactive components of the genetic variance due to editing. Furthermore, the passive genotype and phenotype selection of farm animals is evolving to a “smart” operation by including simulation. Gene expression programming may be mathematically modeled by an Artificial Intelligence approach. Therefore, the Bioinformatics that features the integration of multi-disciplinary technologies of biology, mathematics, and computer science should play an important role in promoting a better understanding of GE and NBT. The application of NBT to improve the sustainability of pig husbandry is certainly too important to be left to geneticists alone, genomic designing of next generation of pigs or any other farm animal should be the result of a partnership between government and industry that supports and values production, people, and the planet.

## CONCLUSION

Genome/gene-editing is a useful NBT methodology that contributes to agrobiodiversity and the realization of a more sustainable food system by achieving greater resistance to disease and climate change while ensuring affordable solutions for farmers and consumers. NBT could also be broadly applied to pig production for purposes such as increasing disease tolerance or resistance and enhancing animal welfare. Currently, the trend of global regulations or legislation for GE organisms is to exempt them from classification as GMOs, but relevant regulatory frameworks remain to be established in most countries. Suitable regulations or legislation for the scientific application of GE that are based on science and comparison with wild counterpart organisms will be a necessary, and include a wise strategy for human development, and the global valorization and commercialization of these organisms can be foreseen. The integration and convergence with digital technologies should play an important role in promoting a better understanding of GE and NBT. Next generation of pig breeding should be smart and sustainable.

## Figures and Tables

**Table 1:** Application of gene-editing (GE) in pigs for animal production

Year	Authors	Target gene	GE method	KO or KI	Achievement	Reference
2015	Qian et al	*MSTN*	ZFN	KO	Improved Meishan pig meat growth through double muscling	[43]
2015	Wang et al	*MSTN*	CRISPR/Cas9	KO	8 stillbirths or early deaths in Landrace piglets, with 2 showing double muscling	[44]
2016	Wang et al	*MSTN*	CRISPR/Cas9 ssODN	KI	Generation of one early dead Large White piglet with a point mutation (c.938G>A)	[45]
2017	Wang et al	*MSTN*	CRISPR/Cas9	KO	Generation of 23 Erhualian pigs with obvious muscular protrusion, wider backs and fuller hips compared with the wild-type control.	[46]
2019	Zou et al	*MSTN*	CRISPR/Cpf1-assisted ssODN	KO	Two heterozygous Durocs with the Belgian Blue mutation	[47]
2020	Li et al	*MSTN*	CRISPR/Cas9	Ed	Introduction of two mutations (PVD20H and GP19del) in the MSTN signal peptide region in Liang Guang Small Spotted pigs, resulting in enhanced muscle mass.	[49]
2018	Xiang et al	*IGF2*	CRISPR/Cas9	Ed	The IGF2 intron 3–3072 site was mutated with abolished repressor binding, the F1 Bama pigs grew faster with normal meat quality.	[48]
2021	You et al	*Fat-1/IGF-1*	CRISPR/Cas9	KI	KI Fat-1 and IGF-1 gene in the Rosa26 locus could increase pork ω-3 PUFA content and decrease the ω-6 PUFA/ω-3 PUFA ratio	[68]
2018	Zou et al	*FBXO40*	CRISPR/Cas9	KO	Simultaneous KI with a Neo resistance selection marker, increasing muscle mass growth by 4% without detectable pathological effects.	[51]
2017	Zhang et al	*UCP1*	CRISPR/Cas9	KI	Increased thermogenesis of piglets, improving survival rate and welfare	[57]
2021	Gu et al	*PPARγ*	CRISPR/Cas9	KI	Intramuscular fat was increased with normal carcass lean ratio	[69]

KO, gene knockout; KI, DNA fragment or exogene knock-in; *MSTN*, myostatin; ZFN, zinc finger nuclease; CRISPR/Cas9, clustered regularly interspaced short palindromic repeat)/CRISPR-associated (Cas) endoribonuclease 9; Cpf1, type V Cas9; ssODN, single strain oligo-DNA; Ed, editing; *IGF*, insulin-like growth factor; *Fat-1*, fatty acid desaturase; PUFA, polyunsaturated fatty acid; *FBXO40*, F-box protein 40; *UCP1*, uncoupling protein 1; *PPARγ*, peroxisome proliferator-activated receptor gamma.

**Table 2:** Studies of CRISPR/Cas9 gene-editing for disease resistance in pigs

Year	Authors	Virus	Targeting gene	KO/Indel	Achievement and conclusion	Reference
2013	Prather et al	PRRSV	CD169	HR	CD169 KO pigs were unresistant to PRRSV infection	[82]
2014	Whitworth et al	PRRSV	CD163	KO	Generation of CD163 KO pigs	[70]
2016	Whitworth et al	PRRSV	CD163	KO	No fever or lung pathogenesis after PRRSV challenge	[84]
2017	Whitworth et al	PRRSV	CD163	KO	CD163 KO sows showed normal pregnancy	[86]
2017	Burkard et al	PRRSV	CD163	Exon 7	Challenge of both PAMs and PMMs with PRRSV genotype 1, subtypes 1, 2, and 3 and PMMs with PRRSV genotype 2 revealed complete resistance to viral infections assessed by replication.	[71]
2018	Burkard et al	PRRSV	CD163	Exon 7	Scavenger receptor cysteine-rich domain 5 (SRCR5) region-deleted pigs were fully resistant to virus infection.	[85]
2018	Yang et al	PRRSV	CD163	KO	CD163 knockout conferred full resistance to highly pathogenic PRRSV infection in pigs without impairing the biological function associated with the gene.	[72]
2019	Guo et al	PRRSV	CD163	Exon 7	Partial SRCR5 region-deleted pigs were completely resistant to PRRSV 2 infection, but PAM still exhibited a cytokine response.	[73]
2019	Wang et al	PRRSV	CD163	Exon 7	Challenged with a highly pathogenic PRRSV strain, the CD163E7D pigs exhibited mild clinical symptoms and had decreased viral loads in blood.	[74]
2021	Tanihara et al	PRRSV	CD163	KO	Transfer of GE vectors via electroporation into in vitro-fertilization zygotes generated one piglet carrying a 5 bp deletion in CD163	[76]
2017	Popescu et al	ASF	CD163	KO	No resistance upon challenging with the ASF virus isolate Georgia 2007/1.	[78]
2013	Lillico et al	ASF	RELA	KO	Generation of live pigs with RELA KO by TALEN and ZFN	[90]
2016	Lillico et al	ASF	RELA	Ed	Interspecies substitution of 3 AA of RELA from warthog to domestic pig by ZFN	[77]
2020	McCleary et al	ASF	RELA	Ed	Substitution of 3 AA of RELA by editing in pigs was not sufficient to confer resilience to ASFV	[91]
2019	Whitworth et al	TGEV/ PEDV	APN	KO	ANPEP null pigs were not susceptible to TGEV infection but retained susceptibility to PEDV infection.	[79]
2019	Tu et al	PEDV	CMAH	KO	CMAH KO piglets with null NGNA expression were not immune to PEDV but may show lessened severity.	[65]
2020	Xu et al	PRRSV TGEV	CD163 pAPN	Exon 7 KO	Double KO pigs were resistant to type II PRRSV and TGEV infection; upon TGEV infection, WT pigs showed pathogenesis but no significant difference in weight gain from dKO pigs.	[75]
2018	Xie et al	CSFV	shRNA	KI	Small hairpin RNA KI in the porcine Rosa26 locus improved resistance to CSFV infection.	[80]

CRISPR/Cas9, clustered regularly interspaced short palindromic repeat)/CRISPR-associated (Cas) endoribonuclease 9; KO, gene knockout; Indel, insertion and deletion; PRRSV, porcine reproductive and respiratory syndrome virus; CD163E7D, CD163 exon 7 deleted; ASF, African swine fever; RELA, p65, v-rel reticuloendotheliosis viral oncogene homolog A; Ed, editing; TGEV, transmissible gastroenteritis virus; PEDV, porcine epidemic diarrhea; CMAH, CMP-N-glycolylneuraminic acid hydroxylase; NGNA, N-glycolylneuraminic acid; pAPN, porcine aminopeptidase N; CSFV, classical swine fever virus; KI, DNA fragment or exo-gene knock-in.
